# Cobalt nanoparticles protect against endocrine disruption in response to multiple abiotic stresses in fish

**DOI:** 10.3389/fimmu.2026.1844641

**Published:** 2026-06-10

**Authors:** Neeraj Kumar, Paritosh Kumar, S.A. Kochewad, Prem Kumar, Dilip Kumar Singh, Kotha Sammi Reddy

**Affiliations:** 1 Indian Council of Agricultural Research (ICAR)-National Institute of Abiotic Stress Management, Pune, India; 2 Indian Council of Agricultural Research (ICAR)-Central Institute of Fisheries Education, Mumbai, India

**Keywords:** climate change, endocrine disruption, fish, milt characteristics, pollution, reproductive efficiency

## Abstract

**Background:**

Climate change, pollution, and deteriorating water quality have emerged as major threats to aquaculture and fish reproduction. *Anabas testudineus* were exposed to multiple abiotic stressors such as arsenic, ammonia toxicity, low pH, and elevated temperature, which disrupt endocrine regulation, impair reproductive hormones, reduce sperm quality, and ultimately decrease reproductive efficiency. These stressors adversely affect the hypothalamic-pituitary-gonadal axis, resulting in hormonal imbalance and poor gamete quality of fish. The present investigation aims to protect the fish against endocrine disruption and improve reproductive hormones by using cobalt nanoparticles diets (Co-NPs).

**Methods:**

An experiment was conducted for 115 days in *Anabas testudineus* of five hundred and four (504) with an average weight of 9.54 ± 0.25 g. The experiment was designed with twelve treatments in triplicate. The experiment was conducted in the triplicate. Cobalt nanoparticles (Co-NPs) were synthesized using green approach and formulated fish diet at 0.2, 0.4, 0.6 and 0.8 mg kg-1 diet. The arsenic (1/10^th^ LC_50_, 2.05 mg L^-1^), ammonia (1/10^th^ of LC_50_ 2.3), low pH (6.5), and elevated temperature (34 °C) were maintained throughout the experiment for 115 days. The endocrine disruption, reproductive hormones and milt quality were evaluated.

**Results:**

Reproductive hormones in both male and female fish such as gonadotropin-releasing hormone (GnRH), progesterone, 11-keto-testosterone (11-KT), follicle-stimulating hormone (FSH), luteinizing hormone-releasing hormone (LH-RH), vitellogenin (Vt), and estradiol (in females) were significantly improved with Co-NP supplementation at 0.4 and 0.6 mg kg^-^¹ under both control and As+NH_3_+pH+T exposure conditions. Co-NPs supplementation also protects from endocrine disruption in the present study. Furthermore, milt/sperm characteristics were evaluated under multiple abiotic stressors and dietary Co-NP treatments. Milt count, milt motility, straight-line velocity (VSL), curvilinear velocity (VCL), progressive motility, average path velocity, linearity (%), and straightness (%) were substantially enhanced by dietary Co-NPs at 0.4 and 0.6 mg kg^-^¹, with or without stress exposure.

**Conclusions:**

Dietary supplementation of Co-NPs at 0.4 and 0.6 mg kg^-^¹ demonstrated strong potential to improve reproductive efficiency in *A. testudineus* under combined abiotic stress conditions (As+NH_3_+pH+T).

## 1 Introduction

In the era of climate change and pollution, ensuring sustainable practices across aquaculture and allied sectors has become increasingly challenging. Aquatic ecosystems are particularly vulnerable to these environmental disturbances, which subsequently affect all aquatic organisms (1–3). In aquaculture, ammonia toxicity, low pH, arsenic contamination, and high temperature stress are common problems that significantly reduce production and productivity. The combined presence of these stressors creates severe physiological stress for aquatic animals and frequently occurs under practical aquaculture conditions. These stressors are known to adversely affect endocrine function, vitellogenin induction, reproductive hormones, milt characteristics, and overall reproductive efficiency in aquatic species (4–6). Arsenic is a highly toxic metalloid and a potent endocrine-disrupting contaminant that profoundly impairs reproductive physiology by disrupting the hypothalamic–pituitary–gonadal (HPG) axis in vertebrates. It interferes with neuroendocrine signaling, alters gonadotropin secretion, and modulates the synthesis and regulation of sex steroid hormones, thereby affecting reproductive homeostasis. Chronic arsenic toxicity also alters reproductive behavior, endocrine feedback mechanisms, and offspring development, ultimately compromising reproductive success and population sustainability (7). Arsenic exposure disrupts the secretion of hypothalamic gonadotropin-releasing hormone (GnRH), which subsequently affects the release of pituitary gonadotropins luteinizing hormone (LH) and follicle-stimulating hormone (FSH) and impairs gonadal steroidogenesis (8, 9). Furthermore, arsenic reduces sperm count and motility by damaging the ultrastructure of the sperm acrosome and flagellar formation (10). It also disrupts ATP production in sperm by impairing oxidative phosphorylation and glycolysis, ultimately affecting sperm activity in animals and fish (11, 12).

Cobalt (Co) is an essential trace element and a vital component of vitamin B_12_ (cobalamin). It plays critical roles in erythropoiesis, nervous system function, and brain activity (13, 14). Cobalt is also important in cellular metabolism, influencing fatty acid metabolism, DNA synthesis, and energy production. It is required for the activation of several enzymes and for stabilizing molecules that contribute to antioxidant defense mechanisms (15). Additionally, cobalt plays a role in regulating blood glucose levels and modulating enzyme activities (16, 17). Vitamin B_12_ functions as a cofactor for methionine synthase (MS) and methylmalonyl-CoA mutase (MCM). Methionine synthase facilitates methionine production by transferring a one-carbon unit from folate to homocysteine, and it is essential for nucleic acid biosynthesis, which requires methyl groups as a key component (18). Methylmalonyl-CoA mutase participates in the Krebs cycle by catalyzing the conversion of methylmalonyl-CoA to succinyl-CoA (19). Cobalt has several important biological functions and may also enhance reproductive efficiency in fish when supplemented at optimal concentrations. It has been shown to improve growth performance, feed intake, hemoglobin levels, and immunity in fish (20–23). Previous research has reported both beneficial and adverse effects of cobalt in various animals; however, studies on its role in fish reproduction remain limited. Nanostructured cobalt materials (Co-NPs) offer potential advantages over inorganic cobalt due to their higher reactivity and larger surface area. The present study demonstrated biologically synthesized cobalt nanoparticles (Co-NPs), which improved reproductive performance at relatively low dietary inclusion levels (0.4 and 0.6 mg kg^-^¹ diet). This study represents the first report demonstrating the role of dietary cobalt-based nanomaterials in enhancing reproductive performance in fish.

Although arsenic, ammonia, low pH, and high temperature act as endocrine disrupting stressors (EDCs) and alter the overall regulation of reproductive hormones (24, 25), their combined effects are even more detrimental. Hormone receptors play a central role in regulating growth and reproduction, and endocrine disruptors can bind to these receptors (26), exerting agonistic or antagonistic effects that lead to abnormal endocrine function (27). Milt characteristics including milt count, sperm motility, straight-line velocity (VSL), curvilinear velocity (VCL), straightness percentage, and linearity percentage are also negatively affected when fish are exposed to a combination of stressors such as arsenic, ammonia, low pH, and elevated temperature.

*Anabas testudineus* is well known for its excellent flavour, therapeutic properties, and nutritional value (28). It is also an ideal species for investigating endocrine disruption and reproductive efficiency under multiple abiotic stress conditions (29). Therefore, the present study aimed to elucidate the mechanisms by which novel cobalt nanoparticle-based diets enhance reproductive efficiency and provide protection against endocrine disruption induced by combined stresses of arsenic, ammonia, low pH, and high temperature in *A. testudineus*. Notably, this study represents the first report demonstrating the role of cobalt nanoparticles in improving reproductive performance and mitigating endocrine disruption in fish.

## 2 Materials and methods

### 2.1 Ethics statement

The care and treatment of animals used in this study adhered to the guidelines of the Committee for Control and Supervision of Experiments on Animals (CCSEA), Ministry of Environment & Forests, Animal Welfare Division, Government of India. The Aquaculture Wet Laboratory at ICAR-NIASM is registered under CCSEA with registration number 2190/GO/RReBi/SL/2022. The experimental protocol for the present study was approved by the Institutional Animal Ethics Committee (IAEC) under approval number NIASM/IAEC/2024/07. All methodologies and experimental procedures strictly complied with the Animal Research: Reporting of *In Vivo* Experiments (ARRIVE) guidelines.

### 2.2 Experimental animal and design

Five hundred and four (504) unmatured *Anabas testudineus* with an average weight of 9.54 ± 0.25 g were selected for the present investigation. Although, the seven male and seven female were kept in one replicate however, forty-two fish were used for one treatment. The experiment was conducted in the triplicate. The fish were obtained from the NIASM farm pond. Prior to the experiment, the fish were acclimated and quarantined for two weeks using a 1% salt dip solution and potassium permanganate (KMnO_4_) treatment. The experiment was conducted in rectangular plastic tanks with a water capacity of 150 L for a duration of 115 days. The study followed a completely randomized design (CRD) comprising twelve treatments, each with three replicates. Details of the treatments are provided in **Table 1**.

**Table 1:** Experimental design of present investigation.

S. no	Details of the treatments	Exposure condition	Diets	Notation
1	Control	No exposure	Control diet	Ctr
2	Exposure to arsenic	As (1/10^th^ of LC_50_ 2.05)	Control diet	As
3	Concurrent exposure to arsenic and low pH	As (1/10^th^ of LC_50_ 2.05) and low pH (6.5)	Control diet	As+pH
4	Concurrent exposure to arsenic, low pH, ammonia and high temperature and fed with control diet	As (1/10^th^ of LC_50_ 2.05), low pH (6.5), NH_3_ (1/10^th^ of LC_50_ 2.3) and high temperature (34 °C)	Control diet	As+pH+NH_3_+T
5	Group fed with Cobalt nanoparticles at 0.2 mg kg-1 diet	No exposure	Cobalt nanoparticles at 0.2 mg kg-1 diet	Co-NPs at 0.2 mg kg^-1^ diet
6	Group fed with Cobalt nanoparticles at 0.4 mg kg-1 diet	No exposure	Cobalt nanoparticles at 0.4 mg kg-1 diet	Co-NPs at 0.4 mg kg^-1^ diet
7	Group fed with Cobalt nanoparticles at 0.6 mg kg-1 diet	No exposure	Cobalt nanoparticles at 0.6 mg kg-1 diet	Co-NPs at 0.6 mg kg^-1^ diet
8	Group fed with Cobalt nanoparticles at 0.8 mg kg-1 diet	No exposure	Cobalt nanoparticles at 0.8 mg kg-1 diet	Co-NPs at 0.8 mg kg^-1^ diet
9	Group concurrently exposed to arsenic, low pH, ammonia and high temperature and fed with cobalt nanoparticles at 0.2 mg kg^-1^ diet	As (1/10^th^ of LC_50_ 2.05), low pH (6.5), NH_3_ (1/10^th^ of LC_50_ 2.3) and high temperature (34 °C)	Cobalt nanoparticles at 0.2 mg kg-1 diet	Co-NPs at 0.2 mg kg^-1^- As+pH+NH_3_+T
10	Group concurrently exposed to arsenic, low pH, ammonia and high temperature and fed with cobalt nanoparticles at 0.4 mg kg^-1^ diet	As (1/10^th^ of LC_50_ 2.05), low pH (6.5), NH_3_ (1/10^th^ of LC_50_ 2.3) and high temperature (34 °C)	Cobalt nanoparticles at 0.4 mg kg-1 diet	Co-NPs at 0.4 mg kg^-1^- As+pH+NH_3_+T
11	Group concurrently exposed to arsenic, low pH, ammonia and high temperature and fed with cobalt nanoparticles at 0.6 mg kg^-1^ diet	As (1/10^th^ of LC_50_ 2.05), low pH (6.5), NH_3_ (1/10^th^ of LC_50_ 2.3) and high temperature (34 °C)	Cobalt nanoparticles at 0.6 mg kg-1 diet	Co-NPs at 0.6 mg kg^-1^- As+pH+NH_3_+T
12	Group concurrently exposed to arsenic, low pH, ammonia and high temperature and fed with cobalt nanoparticles at 0.8 mg kg^-1^ diet	As (1/10^th^ of LC_50_ 2.05), low pH (6.5), NH_3_ (1/10^th^ of LC_50_ 2.3) and high temperature (34 °C)	Cobalt nanoparticles at 0.8 mg kg-1 diet	Co-NPs at 0.8 mg kg^-1^- As+pH+NH_3_+T

### 2.3 Experimental condition

Sodium arsenite (NaAsO_2_) was used as the source of arsenic. The 96 h LC_50_ for arsenic toxicity, determined in the present study, was 20.50 mg L^-^¹, and 1/10^th^ of this concentration was used for the experiment. Stock solutions of arsenic and ammonium chloride (NH_4_Cl) were prepared at 100 mg L^-^¹. Ammonia stress was maintained using ammonium chloride (NH_4_Cl), for which the 96 h LC_50_ was determined as 23.0 mg L^-^¹ in this study (1/10^th^ of LC_50_ of NH_4_Cl as 2.3 mg L^-1^). The pH of the experimental water was maintained at 6.5 using 0.1 N HCl or 0.1 N NaOH, along with a phosphate buffer (0.1 M, pH 6.5), to ensure consistent pH levels throughout 115 days experiment. pH was monitored three times daily using a digital pH meter (29, 30). Water temperature was maintained at 34 °C using a thermostatic heater.

### 2.4 Cobalt-nanoparticles synthesis using biological approaches

#### 2.4.1 Preparation of fish tissue extract

Fish gills were used for the synthesis of Co-NPs. Fish were anesthetized with clove oil (50 mg L^-1^) in emersion method technique. The gill tissues were carefully collected. The tissues were thoroughly cleaned under running water to remove blood and other debris. After homogenization, the supernatant was obtained by centrifuging the tissue homogenate using a tissue homogenizer (Omni Tissue Master Homogenizer, Kennesaw, GA) at 5000–6000 rpm. The resulting supernatant was then filtered through Whatman filter paper (0.45 µm pore size) to obtain the gill extract (3, 31).

#### 2.4.2 Preparation and characterization of cobalt nanoparticles

**Figure 1:**
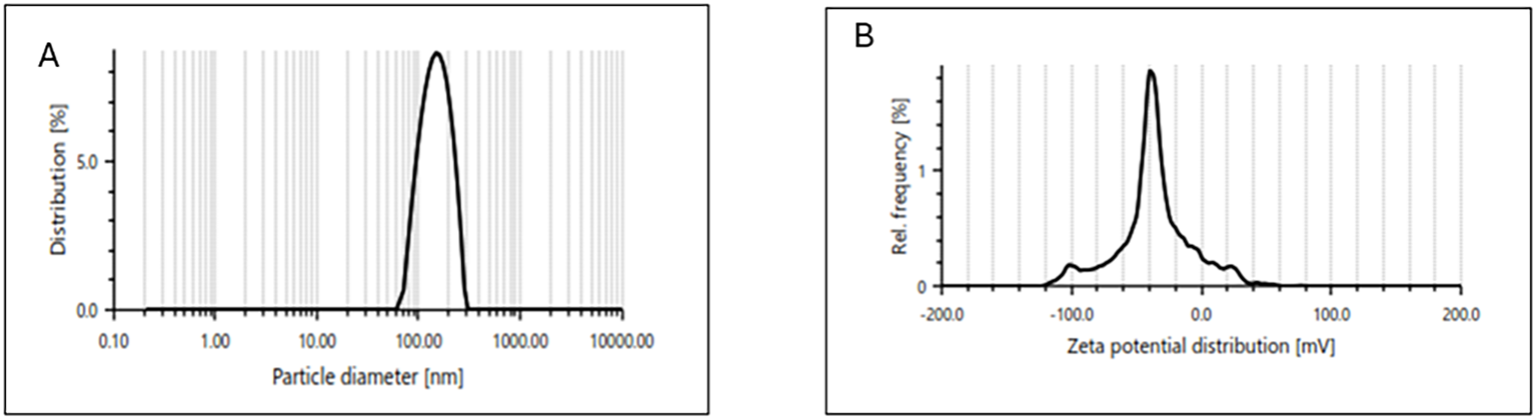
Cobalt-nanoparticles (Co-NPs) **(A)** Size (151 nm) and **(B)** Zeta potential (-39.9 mV).

Cobalt chloride (CoCl_2_) was used for the synthesis of Co-NPs. A solution of cobalt chloride (25 mL, 1 M) and 2 mL of double-distilled water was mixed with 20 mL of fish gill extract under vigorous stirring. The mixture was placed on a magnetic stirrer at 70-80 °C until a visible colour change occurred (after approximately 30–35 minutes). The reaction mixture was then centrifuged at 9000-10, 000 rpm for 15–20 minutes. The supernatant was discarded, and the pellet containing Co-NPs was collected. The pellet was washed three times with ethanol to remove impurities and dried in an oven at 60 °C. The dried powder was subsequently calcined at 100 °C overnight. The synthesized Co-NPs were dispersed in Milli-Q water and subjected to particle size analysis using a particle analyzer (Litesizer 500, Anton Paar, Austria). The mean particle size and zeta potential of the synthesized Co-NPs were 151.2 nm and -39.9 mV, respectively (**Figures 1A, B**).

### 2.5 Feed formulation and feeding practices

Five iso-nitrogenous (35% crude protein) and iso-caloric diets were formulated by incorporating cobalt nanoparticles (Co-NPs) at graded levels of 0, 0.2, 0.4, 0.6, and 0.8 mg kg^-^¹ of diet. Dose standardization of Co-NPs was performed prior to the commencement of the experiment. Based on the range finding study conducted for dose standardization of Co-NPs 0.2, 0.4, 0.6, and 0.8 mg kg^-^¹ diet were chosen for the present study. Locally available feed ingredients were used for diet preparation, and their composition is presented in **Table 2** (32). Gross energy of the diets was calculated following the method of Halver (33). Fish were fed the experimental diets twice daily at 9:00 AM and 5:00 PM. Faecal matter and uneaten feed were removed daily by siphoning. Water quality parameters were monitored regularly following APHA (34) guidelines and were maintained within suitable ranges for the culture of *Anabas testudineus* (29). Approximately two-thirds of the water in each tank were replaced on alternate days, and the concentrations of arsenic, ammonia, and low pH were maintained accordingly.

**Table 2:** Ingredients composition and proximate analysis of experimental diets (% dry matter) of cobalt nanoparticles fed to *Anabas testudineus* for 115 days.

Ingredients	Control	Cobalt nanoparticles (Co-NPs)			
		Co-NPs-0.2	Co-NPs-0.4	Co-NPs-0.6	Co-NPs-0.8
Soybean meal	35.5	35.5	35.5	35.5	35.5
Fish meal	25	25	25	25	25
Groundnut meal	10	10	10	10	10
Wheat flour	17.47	17.4698	17.4696	17.4694	17.4692
Sunflower oil	4.5	4.5	4.5	4.5	4.5
Cod liver oil	1.5	1.5	1.5	1.5	1.5
CMC	2	2	2	2	2
Vitamin and mineral mix*	2	2	2	2	2
Vitamin C	0.03	0.03	0.03	0.03	0.03
Lecithin	2	2	2	2	2
Co-NPs	0	0.0002	0.0004	0.0006	0.0008
	100.00	100.00	100.00	100.00	100.00
Proximate composition (%)					
Crude protein (CP)	35.01 ± 0.02	35.09 ± 0.57	34.97 ± 0.48	35.11 ± 0.03	35.02 ± 0.08
Ether extract (EE)	9.04 ± 0.02	8.95 ± 0.11	9.12 ± 0.04	8.84 ± 0.09	9.08 ± 0.05
Total carbohydrate (TC)	38.11 ± 0.07	37.91 ± 0.59	37.92 ± 0.39	38.35 ± 0.16	37.88 ± 0.06
Organic matter (OM)	90.43 ± 0.06	90.52 ± 0.20	90.40 ± 0.13	90.40 ± 0.27	89.98 ± 0.04
Dry matter (DM)	91.34 ± 0.02	91.30 ± 0.04	90.93 ± 0.43	90.52 ± 0.26	91.05 ± 0.37
Digestible energy (DE)	355.77 ± 0.29	354.65 ± 0.84	355.42 ± 1.64	355.71 ± 0.80	355.16 ± 1.43
Cobalt (Co) (mg kg^-1^ diet)	0.3 ± 0.02	0.54 ± 0.034	0.78 ± 0.041	0.92 ± 0.03	1.12 ± 0.04

a Procured from local market, ^b^Himedia Ltd, Himedia Ltd, ^c^SD Fine Chemicals Ltd., India.

* Manual prepared Vitamin mineral mixture; Composition of vitamin mineral mix (quantity/250 g starch powder): vitamin A 55, 00, 00 IU; vitamin D3 11, 00, 00 IU; vitamin B1:20 mg; vitamin E 75 mg; vitamin K 1, 00 mg; vitamin B12 0.6 mcg; calcium pantothenate 2, 50 mg; nicotinamide 1000 mg; pyridoxine: 100 mg; Zn 500 mg; I 1, 00 mg; Mn: 100 mg, Cu 200 mg; Fe: 750 mg; Ca 50 g; P 30 g; Se: 2 ppm.

Digestible energy (DE) (Kcal/100 g) = (% CP × 4) + (% EE × 9) + (TC × 4).

Data expressed as mean ± SE, n = 3.

### 2.6 Reproductive hormones

The concentrations of gonadotropin-releasing hormone (GnRH) (ELISA Kit: SL0043FI), progesterone (ELISA Kit: S20054FI), 11-keto-testosterone (11-KT) (ELISA Kit: SLD0108FI), and estradiol (ELISA Kit: SL0033FI) were measured using ELISA kits procured from Biostring Company, 633 Napolean, Johnstown, PA-15901, USA. Other hormone assays, including follicle-stimulating hormone (FSH) (GENLISA, KLF0039), luteinizing hormone-releasing hormone (LH-RH) (BYabscience, BY-EF960957), and vitellogenin (Vt) induction (Biosense, USA; Catalog No. V01003402), were also performed. All protocols and procedures were followed as per the instructions provided by the respective manufacturers. All hormone estimations using ELISA were performed based on standard calibration curves provided with each kit. For every assay, a series of known standards supplied by the manufacturer were run in parallel with the samples to generate a standard curve. The concentrations of hormones in fish samples were then calculated by interpolation from these curves. The standard curves were constructed according to the manufacturer’s instructions, and their linearity (or appropriate curve fit, e.g., four-parameter logistic regression) was verified with high correlation coefficients (R² > 0.98).

### 2.7 Milt characteristics

#### 2.7.1 Collection of sperm

Mature male *Anabas testudineus* were anesthetized with clove oil (25 μL L^-^¹), and milt was gently stripped from the abdominal region without sacrificing the fish. The collected milt was placed on a glass plate, kept on ice, and its volume was measured in microliters (µL). Milt samples were then diluted (1:10) in 0.5 mL Eppendorf tubes using a non-activating medium. For activation, the diluted samples were resuspended in distilled water, resulting in a final dilution of 1:100. Subsequently, 1 µL of each activated sample was placed on a glass slide, covered with a cover slip, and immediately examined.

#### 2.7.2 Computer assisted sperm analysis

Milt/Sperm movement was recorded for 2 minutes from the moment of final dilution using a FLIR (GS3-U3-23S8C-C) Motic camera attached to a Leica DM2000 LED microscope with a 40×/0.40 objective lens via an adapter (1280-7921). Image sequences were analyzed with the Sperm Tracker software (Biovis CASA 2000 Motility Plus V4.11, Microptic S.L., Barcelona, Spain), using parameters optimized for fish sperm. Analysis was conducted starting 20 seconds after activation (to allow for focusing and stabilization of fluid motion) and continued for four successive 15-second intervals.

The following parameters were assessed:

1. Milt count
2. Sperm motility (%)
3. Progressive velocity parameters such as VCL: Curvilinear velocity (µm/s)
4. VSL: Straight-line velocity (µm/s)
5. VAP: Average path velocity (µm/s), calculated from the averaged trajectory points over time
6. Linearity (LIN) and other relevant motility characteristics.

### 2.8 Arsenic and cobalt analysis from fish tissues, feed and experimental water

Arsenic concentrations were determined in experimental water as well as in different fish tissues, including liver, muscle, gill, brain, and kidney, across all treatment groups. Additionally, cobalt (Co) content was measured in the fish feeds. Water samples were first filtered through 0.45 µm membrane filters and then acidified by adding 100 µL of concentrated nitric acid (HNO_3_, 69%; Himedia Laboratory Pvt. Ltd., Mumbai, India). Fish tissues and feed samples underwent microwave-assisted digestion using a Multiwave PRO system (Anton Paar GmbH, Austria), employing a digestion mixture of nitric acid and hydrogen peroxide in 5:1 ratio. Once digestion was complete, samples were cooled to room temperature, filtered through Whatman filter paper (0.45 µm pore size), and diluted to a final volume of 50 mL. Multi-element calibration standards were prepared from certified stock solutions and used to generate calibration curves over an appropriate concentration range for each analyte. A minimum of six calibration points were used, and all calibration curves showed excellent linearity (R² ≥ 0.999). The instrument provides low-level detection, with most elements having method detection limits (MDLs) in the 1–10 parts per trillion (ppt or ng/L) range for solutions. Quantification of arsenic in tissues and cobalt in feeds was performed by inductively coupled plasma mass spectrometry (ICP-MS; Agilent 7700 series, Agilent Technologies, USA), following the methodology outlined by Kumar et al. (35, 36).

### 2.9 Statistics

The statistical analysis of experimental data was performed using the Statistical Package for the Social Sciences (SPSS) version 16 software. To assess the normality of the data, the Shapiro-Wilk test was employed, while the Levene test was used to examine the homogeneity of variances. Specifically, after performing analysis of variance (ANOVA), *post hoc* multiple comparison tests (Duncan’s multiple test) were applied with appropriate correction methods to control the treatment wise error rate. These methods adjust the significance threshold to account for multiple testing and reduce the likelihood of false-positive results. A One-Way Analysis of Variance (ANOVA) was conducted, followed by Duncan’s multiple range test for *post-hoc* comparisons. The significance threshold for all tests was set at p<0.05.

## 3 Results

### 3.1 Synthesis of cobalt-nanoparticles using green approach

Co-NPs were synthesized using green approach through fish gill. The size and zetapotential of the Co-NPs were determined as 151 nm and -39.9 mV respectively. The results are presented in **Figure 1**.

### 3.2 Gonadotropin-releasing hormone

**Figure 2:**
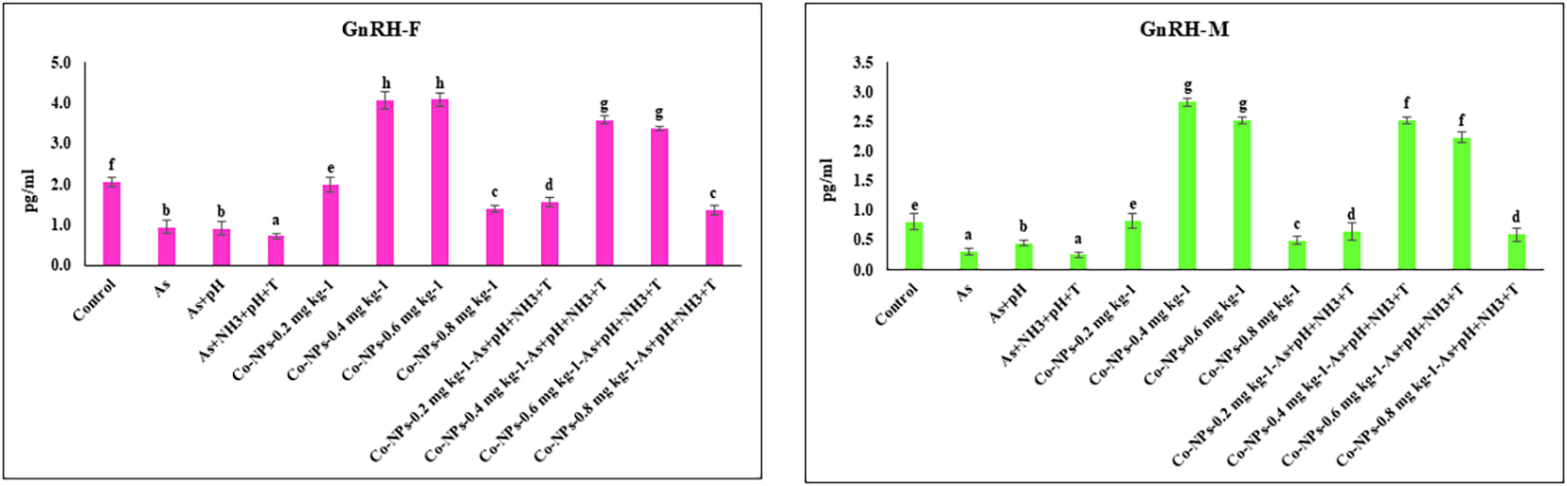
Potential role of cobalt nanoparticles (Co-NPs) in improvement of gonadotropin releasing hormone (GnRH) in female (F) and male (M) of *Anabas testudineus* reared under control and multiple abiotic stress condition for 115 days. Within endpoints and groups, bars with different superscripts differ significantly **(a–h)**. Data expressed as Mean ± SE (n = 4).

GnRH levels in female and male *Anabas testudineus* are presented in **Figures 2**. In females, GnRH decreased significantly (p = 0.0024) under concurrent exposure to arsenic, low pH, ammonia, and high temperature (As+pH+NH_3_+T), followed by the As+pH group and the arsenic-alone group, compared to the control and Co-NPs-supplemented groups. In males, GnRH levels were also significantly reduced in the As+pH+NH_3_+T and arsenic-alone groups, followed by the As+pH group, relative to the control and other treatment groups. Conversely, dietary supplementation with Co-NPs at 0.4 and 0.6 mg kg^-^¹ markedly improved GnRH levels in both female and male *A. testudineus*. Similar improvements were observed in fish exposed concurrently to As+pH+NH_3_+T along with these Co-NPs doses, compared to the control and other treatment groups. However, the lower (0.2 mg kg^-^¹) and higher (0.8 mg kg^-^¹) supplementation levels of Co-NPs were not effective in modulating GnRH levels in either sex of *A. testudineus*.

### 3.3 Progesterone and follicle stimulating hormone

**Figure 3:**
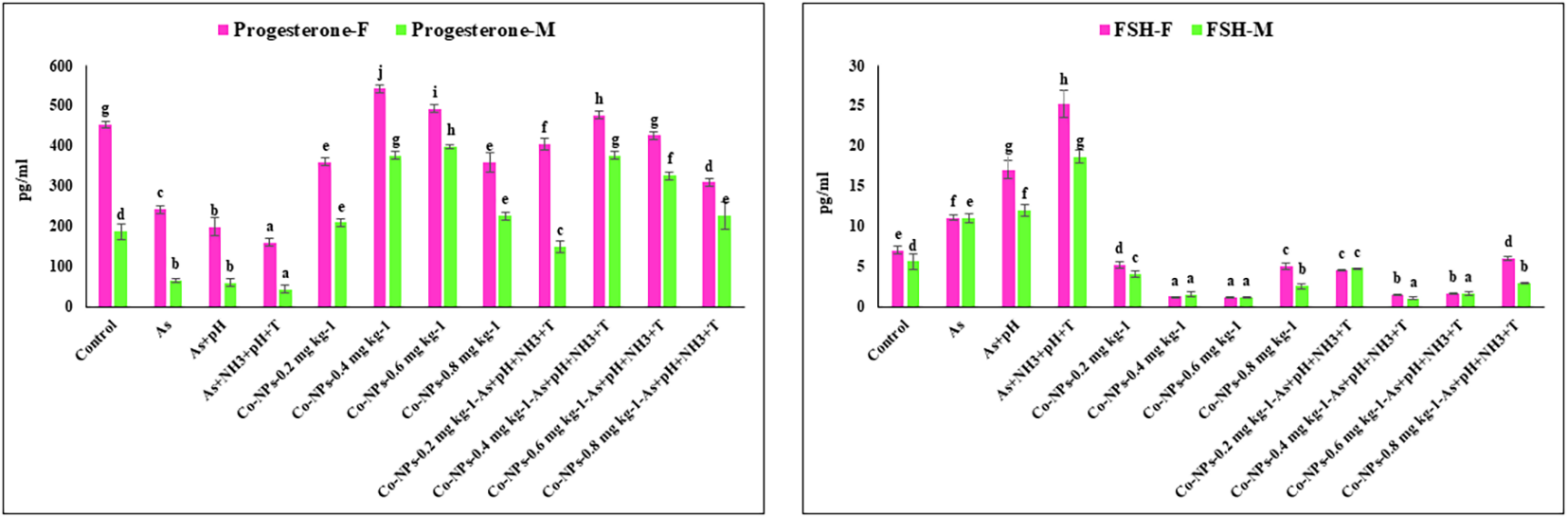
Potential role of cobalt nanoparticles (Co-NPs) in improvement of progesterone and follicle-stimulating hormone (FSH) in female (F) and male (M) of *Anabas testudineus* reared under control and multiple abiotic stress condition for 115 days. Within endpoints and groups, bars with different superscripts differ significantly **(a–j)**. Data expressed as Mean ± SE (n = 4).

The progesterone and FSH levels in female and male *A. testudineus* reared under multiple abiotic stresses (As, As+pH, As+pH+NH_3_+T) were evaluated in this study. Progesterone levels in males (p = 0.001) and females (p = 0.001) were markedly reduced in the As+pH+NH_3_+T group, followed by the As+pH and arsenic alone groups, compared to the control and Co-NPs-supplemented groups. Notably, dietary supplementation with Co-NPs at 0.4 and 0.6 mg kg^-^¹ significantly enhanced progesterone levels in both sexes relative to the control and other treatments. In contrast, FSH levels in females (p = 0.0023) and males (p = 0.0014) were significantly elevated under multiple abiotic stresses, with the highest levels observed in the As+pH+NH_3_+T group, followed by the As+pH and arsenic alone groups, compared to the control and Co-NPs-supplemented groups. Moreover, supplementation with Co-NPs at 0.4 and 0.6 mg kg^-^¹ significantly reduced FSH levels in both sexes, with or without stressor exposure, compared to the control and other treatment groups. The results for progesterone and FSH levels in male and female fish are presented in **Figures 3**.

### 3.4 11-Keto-testosterone and estradiol

**Figure 4:**
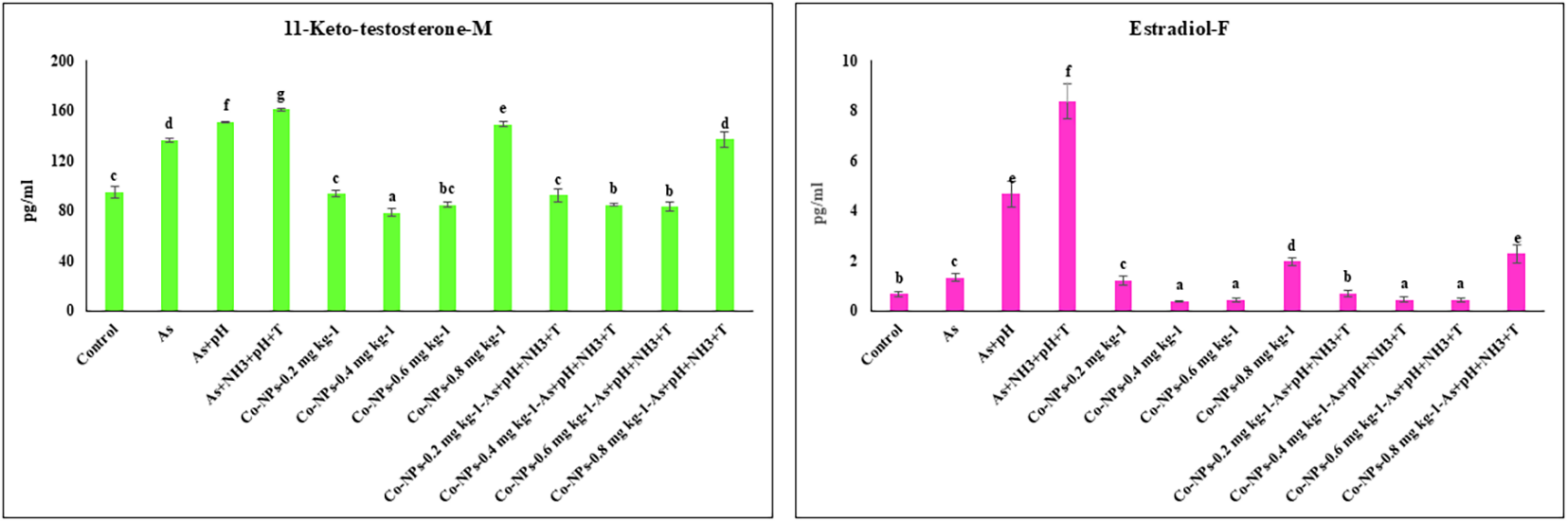
Potential role of cobalt nanoparticles (Co-NPs) in improvement of 11 Keto-testosterones in male and estradiol in female of *Anabas testudineus* reared under control and multiple abiotic stress condition for 115 days. Within endpoints and groups, bars with different superscripts differ significantly **(a–g)**. Data expressed as Mean ± SE (n = 4).

The levels of 11-keto-testosterone (11-KT) in males and estradiol in females of *A. testudineus* were assessed, and the results are presented in **Figures 4**. Both 11-KT (p = 0.0017) and estradiol (p = 0.0012) were markedly elevated in fish exposed to multiple abiotic stresses (As+pH+NH_3_+T), followed by the As+pH group and the arsenic alone group, compared to the control and other treatments. Interestingly, dietary supplementation of Co-NPs at 0.4 mg kg^-^¹, followed by 0.6 mg kg^-^¹ (with or without stressors), significantly reduced 11-KT levels compared to the control and other groups. Similarly, estradiol levels were noticeably reduced in fish fed Co-NPs at 0.4 and 0.6 mg kg^-^¹, irrespective of exposure to multiple abiotic stressors, in comparison with the control and other treatment groups.

### 3.5 Luteinizing hormone-releasing hormone and vitellogenin induction

**Figure 5:**
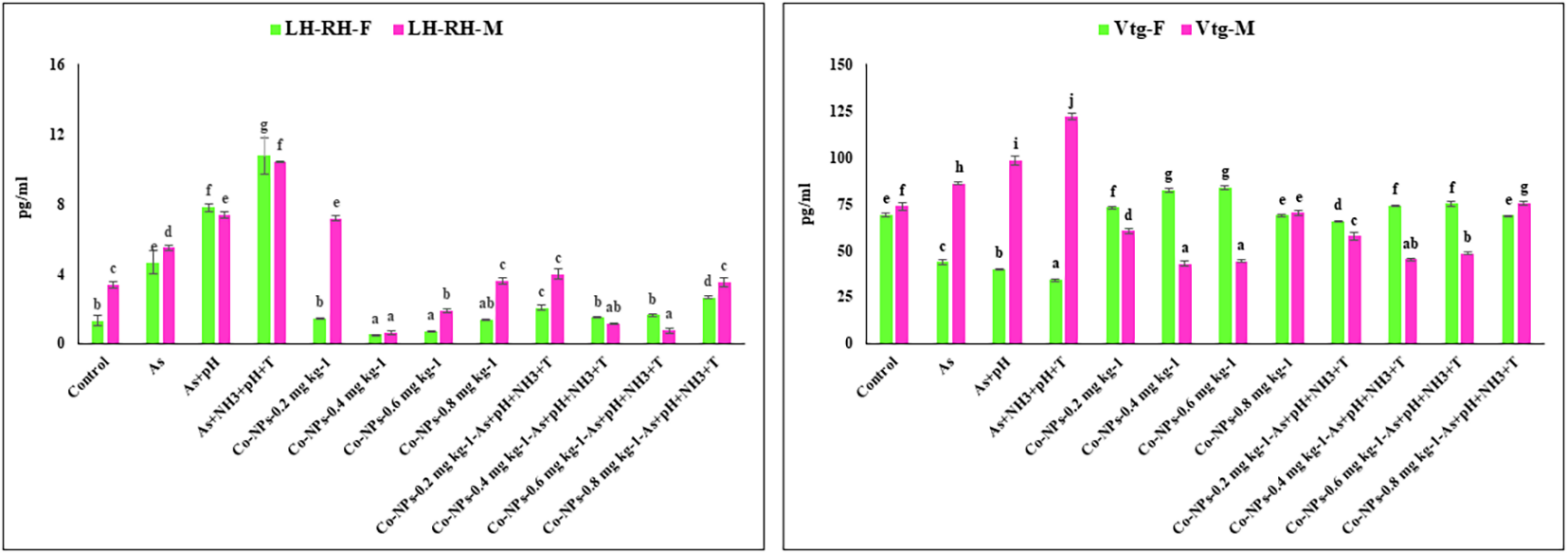
Potential role of cobalt nanoparticles (Co-NPs) in improvement of luteinizing hormone-releasing hormone (LH-RH) vitellogenin induction (Vtg) in female and male of *Anabas testudineus* reared under control and multiple abiotic stress condition for days. Within endpoints and groups, bars with different superscripts differ significantly **(a–j)**. Data expressed as Mean ± SE (n = 4).

LH-RH levels in male and female *A. testudineus* were evaluated, and the results are shown in **Figures 5**. LH-RH levels in both females (p = 0.0001) and males (p = 0.0027) were significantly upregulated under concurrent exposure to arsenic, low pH, ammonia, and high temperature (As+pH+NH_3_+T), followed by the As+pH group and the arsenic-alone group, compared to the control and other treatments. Furthermore, dietary supplementation with Co-NPs at 0.4 and 0.6 mg kg^-^¹, with or without exposure to multiple abiotic stressors, markedly improved LH-RH levels compared to the control and other groups. However, Co-NPs at 0.2 and 0.8 mg kg^-^¹ were not effective in modulating LH-RH levels in *A. testudineus*.

### 3.6 Milt characteristics

#### 3.6.1 Milt count and motility

**Figure 6:**
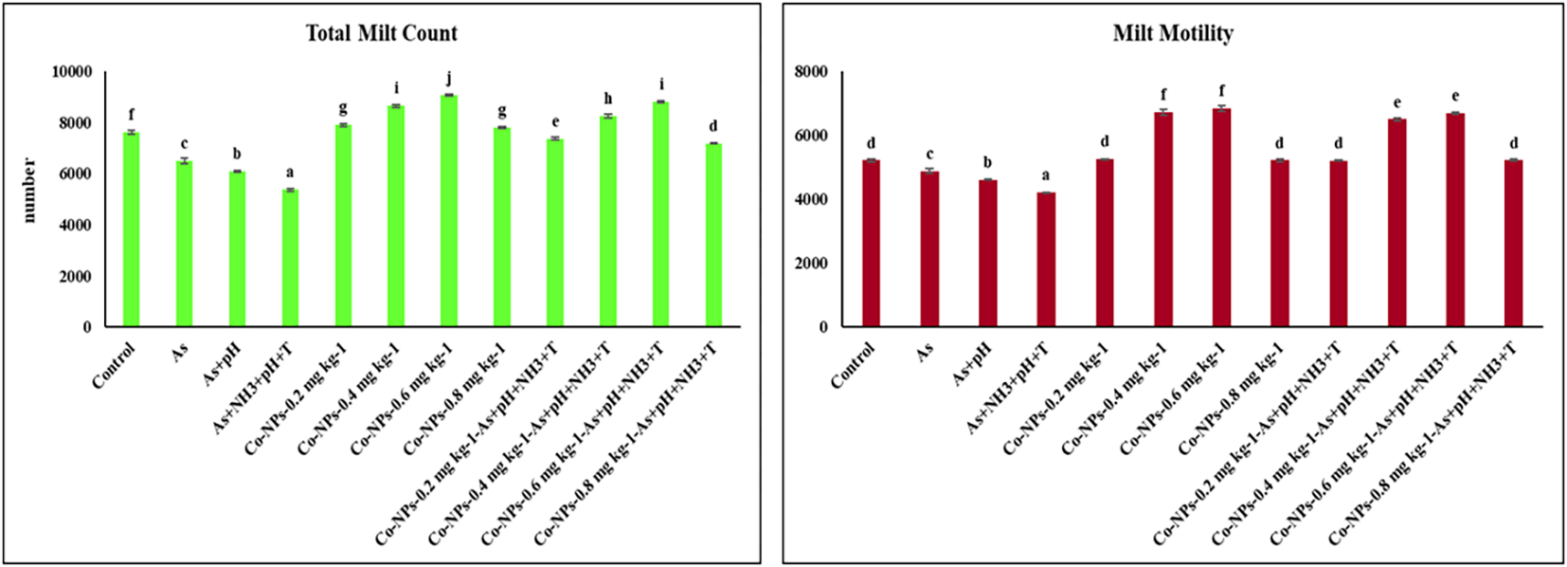
Potential role of cobalt nanoparticles (Co-NPs) in improving total milt count and milt motility of *Anabas testudineus* reared under control and multiple abiotic stress condition for 115 days. Within endpoints and groups, bars with different superscripts differ significantly **(a-j)**. Data expressed as Mean ± SE (n = 3).

*Anabas testudineus* were exposed to arsenic, low pH, ammonia, and high temperature in different combinations and fed Co-NPs-supplemented diets at 0.2, 0.4, 0.6, and 0.8 mg kg^-^¹ for 115 days to evaluate milt characteristics, including milt count and motility. The results are presented in **Figures 6**. Milt count (p = 0.001) and motility (p = 0.001) were significantly reduced under concurrent exposure to As+pH+NH_3_+T, followed by the As+pH and arsenic-alone groups, compared to the control and Co-NPs-supplemented groups. Interestingly, dietary Co-NPs at 0.2, 0.4, 0.6, and 0.8 mg kg^-^¹ without stressors noticeably enhanced the milt count compared to the control and other groups. Furthermore, both milt count and motility were markedly improved with Co-NPs at 0.4 and 0.6 mg kg^-^¹ under stressor exposure compared to other treatments.

#### 3.6.2 Straight line velocity and curvilinear velocity

**Figure 7:**
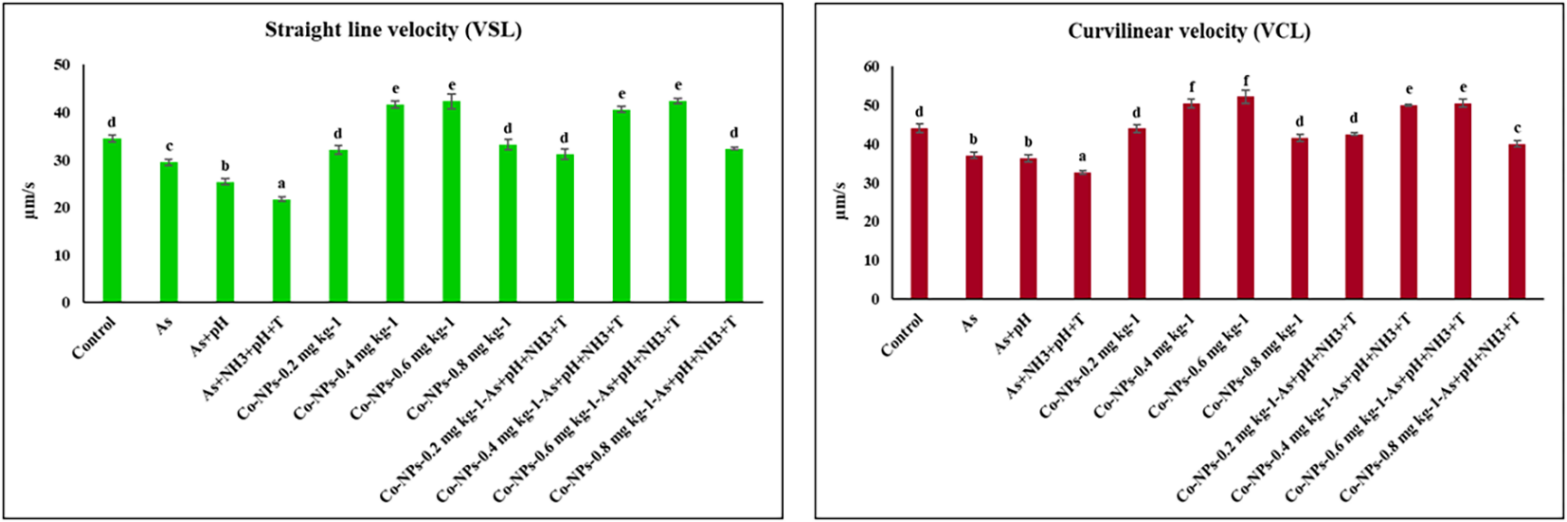
Potential role of cobalt nanoparticles (Co-NPs) in improving VSL and VCL in *Anabas testudineus* reared under control and multiple abiotic stress condition for 115 days. Within endpoints and groups, bars with different superscripts differ significantly **(a–f)**. Data expressed as Mean ± SE (n = 3).

The results for VSL and VCL are presented in **Figures 7**. Concurrent exposure to As+pH+NH_3_+T, followed by As+pH and arsenic alone, significantly reduced VSL (p = 0.0024) and VCL (p = 0.00031) compared to the control and other groups. Supplementation with Co-NPs at 0.4 and 0.6 mg kg^-^¹ noticeably enhanced both VSL and VCL, with or without multiple abiotic stressors, compared to the control and other treatments.

#### 3.6.3 Milt progressive, average path velocity, linearity % and straightness %

**Figure 8:**
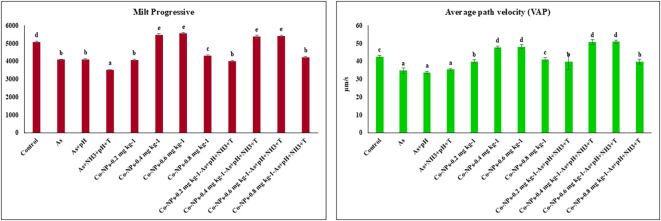
Potential role of cobalt nanoparticles (Co-NPs) in improving milt progressive and average path velocity *Anabas testudineus* reared under control and multiple abiotic stress condition for 115 days. Within endpoints and groups, bars with different superscripts differ significantly **(a–e)**. Data expressed as Mean ± SE (n = 3).

**Figure 9:**
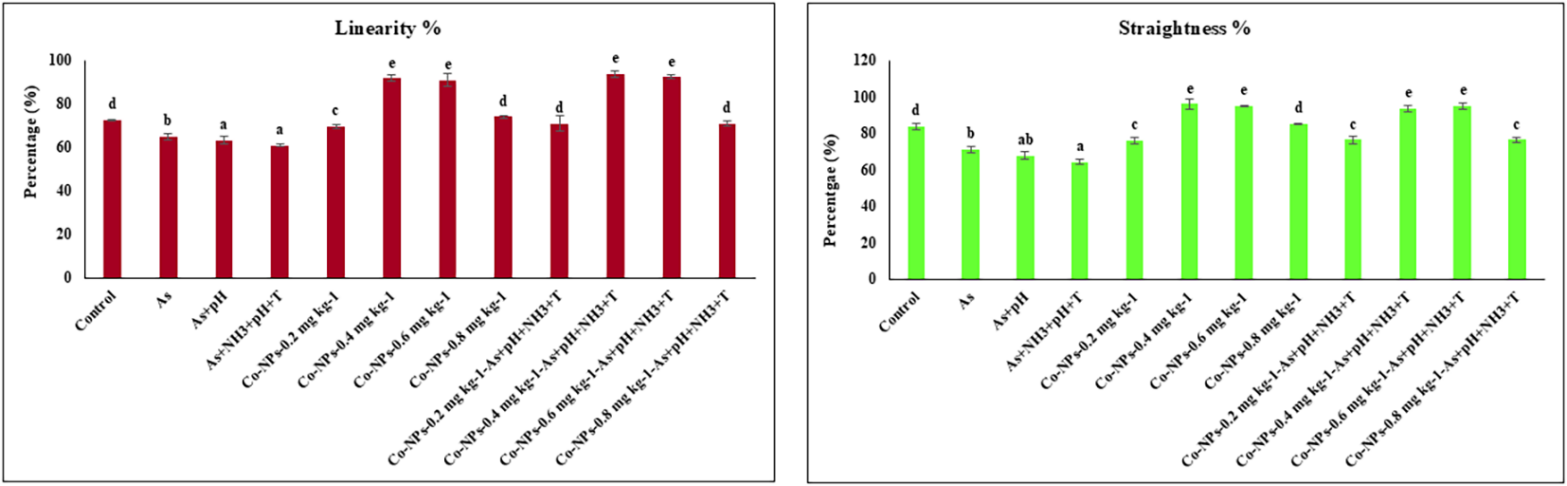
Potential role of cobalt nanoparticles (Co-NPs) in improvement of linearity % and straightness % in *Anabas testudineus* reared under control and multiple abiotic stress condition for 115 days. Within endpoints and groups, bars with different superscripts differ significantly **(a–e)**. Data expressed as Mean ± SE (n = 3).

Exposure to arsenic, low pH, ammonia, and high temperature (As+pH+NH_3_+T), as well as As+pH and arsenic alone, significantly reduced milt progression (p = 0.02), VAP (p = 0.0021), linearity (%) (p = 0.017), and straightness (%) (p = 0.022) in *A. testudineus* compared to the control and Co-NPs-supplemented groups (**Figures 8**, **9**). Notably, dietary supplementation of Co-NPs at 0.4 and 0.6 mg kg^-^¹ markedly improved milt progression, VAP, linearity, and straightness compared to the control and other treatments. However, supplementation at 0.2 and 0.8 mg kg^-^¹ was not effective in modulating milt characteristics, including milt count, motility, VSL, VCL, progression, VAP, linearity, and straightness.

### 3.7 Arsenic detoxification

The arsenic concentration in water and bioaccumulation in different fish tissues such as muscle, liver, gill, kidney and brain tissues is presented in **Table 3**. The concentration of arsenic was higher in groups treated with concurrent exposure to arsenic, low pH, ammonia and high temperature stress followed by As+pH and As alone groups compared to control and other supplemented groups. Moreover, group treated with Co-NPs at 0.6 mg kg^-1^ diet followed by 0.4 mg kg^-1^ diet was lowest arsenic bioaccumulation in muscle tissues compared to other groups. Similarly, the highest arsenic bioaccumulation was present in liver tissue followed by kidney and gill tissues.

**Table 3:** Effect of dietary cobalt nanoparticles (Co-NPs) on detoxification of arsenic in water and different fish tissues reared under control and multiple stress conditions.

Treatments	Water (µg L^-1^)	Muscle (mg kg^-1^)	Liver (mg kg^-1^)	Gill (mg kg^-1^)	Kidney (mg kg^-1^)	Brain (mg kg^-1^)
Ctr	ND	ND	ND	ND	ND	ND
As	800.24 ± 3.45	2.48 ± 0.01	6.54 ± 0.35	4.82 ± 0.18	5.74 ± 0.67	1.85 ± 0.014
As+pH	812.69 ± 4.11	3.14 ± 0.03	7.84 ± 0.36	3.84 ± 0.27	6.41 ± 0.16	1.24 ± 0.05
As+NH_3_+pH+T	943.62 ± 6.84	3.85 ± 0.12	8.85 ± 0.11	3.96 ± 0.08	6.89 ± 0.47	1.08 ± 0.01
Co-NPs-0.2 mg kg^-1^ diet	ND	ND	ND	ND	ND	ND
Co-NPs-0.4 mg kg^-1^ diet	ND	ND	ND	ND	ND	ND
Co-NPs-0.6 mg kg^-1^ diet	ND	ND	ND	ND	ND	ND
Co-NPs-0.8 mg kg^-1^ diet	ND	ND	ND	ND	ND	ND
Co-NPs-0.2 mg kg^-1^- As+NH_3_+pH+T	124.52 ± 2.74	0.98 ± 0.014	3.56 ± 0.14	1.85 ± 0.017	3.52 ± 0.06	1.04 ± 0.027
Co-NPs-0.4 mg kg^-1^- As+NH_3_+pH+T	86.48 ± 0.84	0.11 ± 0.012	2.56 ± 0.32	1.12 ± 0.023	1.45 ± 0.021	0.89 ± 0.016
Co-NPs-0.6 mg kg^-1^- As+NH_3_+pH+T	75.48 ± 1.48	0.14 ± 0.01	1.85 ± 0.47	0.85 ± 0.011	1.27 ± 0.013	0.24 ± 0.011
Co-NPs-0.8 mg kg^-1^- As+NH_3_+pH+T	98.47 ± 1.06	1.05 ± 0.019	3.56 ± 0.71	2.18 ± 0.18	4.18 ± 0.018	0.17 ± 0.01

Data expressed as Mean ± SE (n=3).

## 4 Discussion

In the current scenario, climate change and water pollution are severely affecting the development and reproductive efficiency of aquatic organisms, including fish. Successful reproduction of the origin of each new generation depends largely on the proper functioning of reproductive hormones and gamete quality. Stressors such as high temperature, ammonia, arsenic contamination, and low pH conditions in aquaculture systems cause endocrine disruption and impair reproductive performance, including hormonal regulation and milt characteristics (4, 37). The present study addresses these critical issues related to climate change and water pollution by evaluating the mitigating effects of dietary cobalt nanoparticles (Co-NPs. Gonadotropin-releasing hormone (GnRH) levels in male and female *A. testudineus* were markedly reduced under multiple abiotic stress conditions (As+pH+NH_3_+T, As+pH, and As). Since reproductive hormones are regulated through the hypothalamic-pituitary-gonadal (HPG) axis, exposure to these stressors is likely to suppress HPG axis activity by inhibiting GnRH secretion (38–40). In addition, stress-induced endocrine and inflammatory mediators, including pro-inflammatory cytokines, glucocorticoids, and corticotropin releasing hormone, may further contribute to the suppression of GnRH release (41, 42). Interestingly, dietary Co-NPs significantly enhanced GnRH levels under both stress and control conditions. This effect may be attributed to the ability of cobalt to form cobalt-GnRH complexes, which exhibit high binding affinity toward GnRH receptors (43, 44). Blitek et al. (45) also reported that cobalt-GnRH complexes stimulate luteinizing hormone (LH) secretion more effectively than GnRH alone at lower cobalt level. The present findings support this mechanism, where dietary Co-NPs at 0.4 and 0.6 mg kg^-^¹ likely facilitated the formation of Co-NPs-GnRH complexes, enhancing LH release as reflected in this study.

Multiple abiotic stressors significantly reduced progesterone levels while elevating follicle stimulating hormone (FSH) levels in male and female *A. testudineus*. Dietary Co-NPs at 0.4 and 0.6 mg kg^-^¹ markedly increased progesterone levels and reduced FSH level. The hormonal imbalance induced by arsenic, ammonia, low pH, and elevated temperature may be associated with disruption of gonadotropin synthesis and release through impairment of hypothalamic regulation, leading to decreased progesterone and elevated FSH levels (46). As discussed earlier, cobalt binding to GnRH has been shown to enhance LH and FSH secretion (45), and cobalt may also stimulate progesterone production, as previously demonstrated in rats (47). A similar endocrine regulatory mechanism may also occur in fish. Furthermore, exposure to arsenic, ammonia, low pH, and high temperature significantly increased 11-keto-testosterone levels in males and estradiol levels in females. However, dietary Co-NPs at 0.4 and 0.6 mg kg^-^¹ effectively reduced the levels of both steroid hormones. Arsenic has a known affinity for male reproductive tissues and can bind to sulfhydryl groups in chromatin and flagellar proteins, disrupting spermatogenesis and oogenesis (48, 49). To the best of our knowledge, this is the first report demonstrating the role of dietary Co-NPs in regulating 11-keto-testosterone and estradiol in *A. testudineus*. This effect may be linked to cobalt’s role as a cofactor in vitamin B_12_ metabolism and its involvement in maintaining normal blood circulation, which may support endocrine regulation and steroidogenesis.

Overall, exposure to multiple abiotic stressors (As+NH_3_+pH+T) induced pronounced endocrine disruption in *A. testudineus*, as evidenced by significant alterations in reproductive hormone profiles. This stress induced hormonal perturbations indicate severe impairment of the hypothalamic-pituitary-gonadal (HPG) axis, which plays a central role in regulating gonadal development, steroidogenesis, and reproductive function in fish. Notably, dietary Co-NPs at 0.4 and 0.6 mgkg^-^¹ markedly improved hormonal homeostasis and mitigated endocrine dysfunction under stress conditions. Although this is the first study to evaluate the endocrine modulatory role of Co-NPs in fish exposed to multiple abiotic stressors, the observed responses are biologically and physiologically plausible. Luteinizing hormone-releasing hormone (LH-RH) levels in male and female fish were significantly elevated under combined stress exposure (As+NH_3_+pH+T), followed by As+pH and arsenic alone. The elevation in LH-RH may represent a compensatory neuroendocrine response to stress induced gonadal dysfunction and impaired steroidogenesis. Increased cortisol levels, as reported in our related study, may exert anti-gonadal effects by disrupting feedback regulation within the HPG axis under arsenic, ammonia, low pH and high temperature stress conditions. Chronic activation of the hypothalamic-pituitary-interrenal (HPI) axis and elevated cortisol are known to interfere with reproductive endocrine signaling, suppress gonadal activity, and alter sex steroid synthesis. Similarly endocrine alterations have previously been reported in rats exposed to environmental toxicants (50), although comparable studies in fish remain limited. Furthermore, elevated LH-RH levels may also reflect dysregulation of testosterone biosynthesis and impaired gonadal feedback mechanisms under multiple stress conditions. Interestingly, dietary supplementation with Co-NPs at 0.4 and 0.6 mg kg^-^¹ significantly reduced LH-RH levels under both stressed and non-stressed conditions, suggesting a potential stabilizing effect of cobalt nanoparticles on neuroendocrine regulation and reproductive hormonal balance.

Vitellogenin (Vtg) induction was significantly reduced in female *A. testudineus* but markedly elevated in males under multiple abiotic stress conditions (As+pH+NH_3_+T, As+pH, and As alone). The decline in female Vtg levels may be attributed to impaired liver vitellogenin synthesis caused by arsenic toxicity, ammonia stress, low pH and high temperature stress, which can disrupt hepatocellular function and estrogen-mediated transcriptional regulation. In contrast, the abnormal induction of Vtg in males suggests endocrine feminization and estrogenic disruption induced by these stressors. Similar alterations in Vtg expression have previously been reported in zebrafish exposed to arsenic (6). Vitellogenin is a yolk precursor phospholipoglycoprotein synthesized primarily in the liver under estrogenic stimulation and subsequently transported to developing oocytes during vitellogenesis (51, 52). Therefore, alterations in Vtg synthesis and accumulation are considered sensitive biomarkers of endocrine disruption and impaired reproductive physiology in fish. The reduced Vtg production observed in females indicates compromised estrogen signaling and defective yolk protein synthesis, which may adversely affect oocyte maturation and reproductive success. Conversely, elevated Vtg induction in males reflects abnormal activation of estrogen-responsive pathways and disruption of normal sex-specific endocrine regulation. Interestingly, dietary Co-NPs appeared to normalize Vtg induction patterns under stress conditions, suggesting a protective role against endocrine and hepatic dysfunction. The beneficial effects of Co-NPs may be associated with improved liver metabolic activity, stabilization of estrogenic signaling pathways, and enhanced protein biosynthesis under stress exposure. In addition, cobalt may indirectly support vitellogenin synthesis by alleviating oxidative stress and preserving hepatocyte integrity. However, to the best of our knowledge, no previous studies have evaluated the role of cobalt nanoparticles in modulating vitellogenin dynamics under multiple abiotic stress conditions in fish.

Milt and sperm quality parameters, including total milt count, sperm motility, curvilinear velocity (VCL), straight-line velocity (VSL), average path velocity (VAP), progressive motility, linearity, and straightness, were significantly impaired in *A. testudineus* exposed to multiple abiotic stressors. These findings indicate severe reproductive dysfunction and compromised spermatogenic activity under combined exposure to (As+NH_3_+pH+T). In contrast, dietary Co-NPs significantly improved all evaluated milt and sperm quality parameters, suggesting a protective role against stress induced reproductive impairment. Arsenic toxicity is known to adversely affect spermatogenesis and sperm physiology through multiple mechanisms, including reduction in spermatozoa production, disruption of mitochondrial energy metabolism, and oxidative damage to sperm membranes (53, 54). Arsenic can bind to sulfhydryl (-SH) groups of sperm proteins, thereby altering protein conformation and enzymatic activity essential for sperm viability and motility. In addition, arsenic induced reactive oxygen species (ROS) generation promotes lipid peroxidation of sperm plasma membranes, resulting in loss of membrane fluidity, structural instability, and impaired motility (55). Multiple stressors may also interfere with acrosome biogenesis and flagellar assembly, ultimately affecting sperm maturation and swimming performance (10). Furthermore, arsenic exposure disrupts cellular respiration and ATP synthesis by impairing mitochondrial oxidative phosphorylation, leading to reduced energy availability for sperm motility and fertilization processes (56, 57). The improvement in sperm quality observed following Co-NPs supplementation may be associated with enhanced cellular metabolism, stabilization of membrane integrity, and protection against oxidative stress. Cobalt is an essential component of vitamin B_12_ (cobalamin) and plays a critical role in several metabolic pathways involved in nucleic acid synthesis, methylation reactions, and mitochondrial energy production. Cobalt-dependent enzymes, including methylmalonyl-CoA epimerase and related metabolic cofactors, are involved in fatty acid and amino acid metabolism, which are essential for maintaining sperm membrane composition and cellular energetics (58, 59). Proper membrane lipid organization is crucial for sperm osmoregulation, membrane fluidity, flagellar function, and motility performance (60, 61). Moreover, dietary Co-NPs at 0.4 and 0.6 mg kg^-^¹ improved arsenic detoxification in different fish tissues. This protective effect may be linked to the regulatory role of cobalt in modulating the expression of apoptosis related genes such as caspases, and cytochrome P450 (*CYP450*) mediated detoxification pathways. Regulation of these molecular pathways may contribute to reduced oxidative injury, enhanced xenobiotic metabolism, and improved cellular survival under multiple abiotic stress conditions.

## 5 Conclusions

The present scenario is witnessed for increasing pollution and climate change, which are affecting all ecosystems, including aquatic system. The reproductive efficiency of aquatic organisms is being severely impacted, resulting in declines in reproductive hormones and milt quality in fish. The present study identifies a suitable nutritional strategy using cobalt nanoparticles (Co-NPs) at different dietary doses to mitigate the effects of arsenic, ammonia, low pH, and high temperature stress in fish. Based on the present investigation, dietary Co-NPs at 0.4 and 0.6 mg kg^-^¹ significantly enhanced the reproductive efficiency of fish reared under multiple abiotic stress conditions. This study is the first to report on role of Co-NPs in improving reproductive performance in fish exposed to multiple abiotic stressors.

## Data Availability

All relevant data is contained within the article. The original contributions presented in the study are included in the article/supplementary material, further inquiries can be directed to the corresponding author.

## Glossary

11-KT: 11-keto-testosterone
NH_3_: Ammonia
ARRIVE: Animal Research: Reporting of *In Vivo* Experiments
As: Arsenic
CMC: Carboxymethyl cellulose
Co-NPs: Cobalt Nanoparticles
Co: Computer assisted sperm analysis (CASA)Cobalt
CCSEA: Control and Supervision of Experiments on Animals
CP: Crude protein
VCL: Curvilinear velocity
EDCs: Endocrine disrupting stressors
EE: Ether extract
FSH: Follicle-stimulating hormone
GnRH: Gonadotropin-releasing hormone
T: High temperature
HPG: Hypothalamic&ndash;pituitary&ndash;gonadal
ICPMS: Inductively Coupled Plasma Mass Spectrometry
IPCC: Intergovernmental Panel on Climate Change
LC_50_: Lethal concentration
LH-RH: Luteinizing hormone-releasing hormone
MS: Methionine synthase
MCM: Methylmalonyl-CoA mutase
PME: Prioritization, Monitoring and Evaluation
RAC: Research Advisory Committee
SGR: Specific growth rate
VSL: Straight-line velocity
TAN: Total ammonia nitrogen
Vt: Vitellogenin

## References

[1] Shinde A Sharma R Kumar P Kumar T Reddy KS Kumar N . Combined effect of mercury and ammonia toxicity and its mitigation through selenium nanoparticles in fish. Aquat Toxicol. (2025) 280:107270. doi: 10.1016/j.aquatox.2025.107270 39954588

[2] Kumar N Chandan NK Bhushan S Singh DK Kumar S . Health risk assessment and metal contamination in fish, water and soil sediments in the East Kolkata Wetlands, India, Ramsar site. Sci Rep. (2023) 13:1546. doi: 10.1038/s41598-023-28801-y 36707609 PMC9883242

[3] Kumar N Thorat ST Gunaware MA Kumar P Reddy KS . Unraveling gene regulation mechanisms in fish: insights into multistress responses and mitigation through iron nanoparticles. Front Immunol Comp Immunol. (2024), 15–2024. doi: 10.3389/fimmu.2024.1410150 38947331 PMC11211354

[4] Qi Q Zhang C Xu R Lv C Xue Y Wang P . High ammonia nitrogen-induced reproductive toxicity in goldfish (Carassius auratus) mature ovary. Aquacult Res. (2024) 9577902:9. doi: 10.1155/2024/9577902 40201959

[5] Santos JAD Soares CM Andréa Bialetzki A . Effects of pH on the incubation and early development of fish species with different reproductive strategies. Aquat Toxicol. (2019) 219:105382. doi: 10.1016/j.aquatox.2019.105382 31865068

[6] Rachamalla M da Silva FC Putnala SK Hecker M Niyogi S . Maternal and paternal dietary arsenic exposure impairs reproduction and development in zebrafish offspring: the role of HPG axis dysregulation and altered DNA methylation. Environ pollut. (2025) 380:126528. doi: 10.1016/j.envpol.2025.126528 40436093

[7] Koysombat K Dhillo WS Abbara A . Assessing hypothalamic pituitary gonadal function in reproductive disorders. Clin Sci. (2023) 137(11):863–79. doi: 10.1042/CS20220146 37272254 PMC10248125

[8] Chen Y Sun Y Zhao A Cai X Yu A Xu Q . Arsenic exposure diminishes ovarian follicular reserve and induces abnormal steroidogenesis by DNA methylation. Ecotoxicol Environ Saf. (2022) 241:113816. doi: 10.1016/j.ecoenv.2022.113816 36068745

[9] Tasci T Eldem V Erkan M . Sodium arsenic alters the gene expression of some steroidogenic genes in TM3 Leydig cell. Celal Bayar Üniversitesi Fen Bilimleri Dergisi. (2019) 15(3):265–70. doi: 10.18466/cbayarfbe.540544

[10] Han Y Liang C Yu Y Manthari RK Cheng C Tan Y . Chronic arsenic exposure lowered sperm motility via impairing ultra-microstructure and key proteins expressions of sperm acrosome and flagellum formation during spermiogenesis in male mice. Sci Tot Environ. (2020) 734:139233. doi: 10.1016/j.scitotenv.2020.139233 32460071

[11] du Plessis SS Agarwal A Mohanty G van der Linde M . Oxidative phosphorylation versus glycolysis: what fuel do spermatozoa use? Asian J Androl. (2015) 17:230–5. doi: 10.4103/1008-682x.135123 25475660 PMC4650467

[12] Tourmente M Villar-Moya P Rial E Roldan ER . Differences in ATP generation via glycolysis and oxidative phosphorylation and relationships with sperm motility in mouse species. J Biol Chem. (2015) 290:20613–26. doi: 10.1074/jbc.m115.664813 26048989 PMC4536464

[13] Perrault JR Buchweitz JP Lehner AF . Essential, trace and toxic element concentrations in the liver of the world’s largest bony fish, the ocean sunfish (Mola mola). Mar pollut Bull. (2014) 79:348–53. doi: 10.1016/j.marpolbul.2013.11.026 24341944

[14] Rizzo G Lagana AS . A review of vitamin B_12_. Mol Nutr. (2020), 105–29. doi: 10.1016/b978-0-12-811907-5.00005-1 38826717

[15] Tiszler M Dobrakowski M Olszak-Wąsik K Machon-Grecka A Bellanti F Kasperczyk S . Cobalt’s role in modulating antioxidant systems and semen quality in males. Reprod Toxicol. (2024) 123:108524. doi: 10.1016/j.reprotox.2023.108524 38104640

[16] Siddiqui K Bawazeer N Scaria Joy S . Variation in macro and trace elements in progression of type 2 diabetes. Sci World J. (2014) 2014:1–9. doi: 10.1155/2014/461591 25162051 PMC4138889

[17] Speich M Pineau A Ballereau F . Minerals, trace elements and related biological variables in athletes and during physical activity. Clin Chim Acta. (2001) 312:1–11. doi: 10.1016/s0009-8981(01)00598-8 11580904

[18] McDowell LR . Vitamins in animal nutrition: comparative aspects to human nutrition. San Diego, CA: Academic Press (1989).

[19] Takahashi-Iñiguez T García-Hernandez E Arreguín-Espinosa R Flores ME . Role of vitamin B12 on methylmalonyl-CoA mutase activity. J Zhejiang Univ-Sci B. (2012) 13:423–37. doi: 10.1631/jzus.b1100329 22661206 PMC3370288

[20] Bogard JR Thilsted SH Marks GC Waha MA Hossain MAR Jakobsen J . Nutrient composition of important fish species in Bangladesh and potential contribution to recommended nutrient intakes. J Food Compos Anal. (2015) 42:120–33. doi: 10.1016/j.jfca.2015.03.002 38826717

[21] Goyal V . Vitamin B12. IOSR J Pharm. (2015) 5:30–5. doi: 10.22214/ijraset.2019.3242

[22] Lukaski HC . Vitamin and mineral status: effects on physical performance. Nutrition. (2004) 20:632–44. doi: 10.1016/j.nut.2004.04.001 15212745

[23] Partearroyo T Ubeda N Montero A Achon M Varela-Moreiras G . Vitamin B12 and folic acid imbalance modifies NK cytotoxicity, lymphocytes B and lymphoprolipheration in aged rats. Nutrients. (2013) 5:4836–48. doi: 10.3390/nu5124836 24288024 PMC3875921

[24] Baroiller JF Guiguen Y Fostier A . Endocrine and environmental aspects of sex differentiation in fish. Cell Mol Life Sci. (1999) 55:910–31. doi: 10.1007/978-3-0348-7781-7_9 11301598

[25] Guerrero-Estévez S Moreno-Mendoza N . Sexual determination and differentiation in teleost fish. Rev Fish Biol Fish. (2010) 20:101–21. doi: 10.1007/s11160-009-9123-4 30311153

[26] Diamanti-Kandarakis E Bourguignon JP Giudice LC Hauser R Prins GS Soto AM . Endocrine-disrupting chemicals: an Endocrine Society scientific statement. Endocr Rev. (2009) 30:293–342. doi: 10.1210/er.2009-0002 19502515 PMC2726844

[27] Zoeller RT Brown TR Doan LL Gore AC Skakkebaek NE Soto AM . Endocrine-disrupting chemicals and public health protection: a statement of principles from The Endocrine Society. Endocrinology. (2012) 153:4097–110. doi: 10.1210/en.2012-1422 22733974 PMC3423612

[28] Helmizuryani MB Khotimah K . Reproduction performance of climbing perch Anabas testudineus F1 and F2 broodstock with different dietary supplementation. J Akua Indonesea. (2018) 17:61–7. doi: 10.19027/jai.17.1.61-67

[29] Kumar N Bhushan S Patole PB Gite A . Multi-biomarker approach to assess chromium, pH and temperature toxicity in fish. Comp Biochem Physiol Part C Toxicol Pharmacol. (2022) 254:109264. doi: 10.1016/j.cbpc.2021.109264 35041967

[30] Kumar N Thorat ST Chavhan SR . Multifunctional role of dietary copper to regulate stress-responsive gene for mitigation of multiple stresses in Pangasianodon hypophthalmus. Sci Rep. (2024) 14:2252. doi: 10.1038/s41598-024-51170-z 38278845 PMC10817903

[31] Rokade A Thorat ST Chandramore K Reddy KS Kumar N . Integrating immunity, antioxidative status, and gene regulation against nickel and high-temperature stress in fish: selenium nanoparticles for mitigation. Environ Sci pollut Res. (2025) 32:3987–4003. doi: 10.1007/s11356-025-35947-x 39843820

[32] AOAC . Official methods of analysis of the association of official analytical chemists. Arlington: AOAC International (1995).

[33] Halver JE . The nutritional requirements of cultivated warm water and cold water fish species. In: Report of the FAO technical conference on aquaculture, kyoto, Japan, 26 may–2 june 1976. FAO fisheries report no. 188 FI/ R188 (En) (1976). Kyoto (Japan) p. 9.

[34] APHA-AWWA-WEF . Standard methods for the estimation of water and waste water. Clesceri LS Greenberg AE Eaton AD , editors. Washington, DC: American Public Health Association, American Water Works Association, Water Environment Federation (1998).

[35] Kumar N Krishnani KK Meena KK Gupta SK Singh NP . Oxidative and cellular metabolic stress of Oreochromis mossambicus as biomarkers indicators of trace element contaminants. Chemosphere. (2017) 171:265–74. doi: 10.1016/j.chemosphere.2016.12.066 28027471

[36] Kumar N Krishnani KK Gupta SK Singh NP . Cellular stress and histopathological tools used as biomarkers in Oreochromis mossambicus for assessing metal contamination. Environ Toxicol Pharmacol. (2017) 49:137–47. doi: 10.1016/j.etap.2016.11.017 27992807

[37] Celino-Brady FT Lerner DT Seale AP . Experimental approaches for characterizing the endocrine-disrupting effects of environmental chemicals in fish. Front Endocrinol (Lausanne). (2021) 11:619361. doi: 10.3389/fendo.2020.619361 33716955 PMC7947849

[38] Matsuwaki T Kayasuga Y Yamanouchi K Nishihara M . Maintenance of gonadotropin secretion by glucocorticoids under stress conditions through the inhibition of prostaglandin synthesis in the brain. Endocrinology. (2006) 147:1087–93. doi: 10.1210/en.2005-1056 16293664

[39] Rivier C Rivier J Vale W . Stress-induced inhibition of reproductive functions: role of endogenous corticotropin-releasing factor. Science. (1986) 231:607–9. doi: 10.1126/science.3003907 3003907

[40] Rivier C Rivest S . Effect of stress on the activity of the hypothalamic-pituitary-gonadal axis: peripheral and central mechanisms. Biol Reprod. (1991) 45:523–32. doi: 10.1095/biolreprod45.4.523 1661182

[41] Watanobe H Hayakawa Y . Hypothalamic interleukin-1b and tumor necrosis factor-a, but not interleukin-6, mediate the endotoxin-induced suppression of the reproductive axis in rats. Endocrinology. (2003) 144:4868–75. doi: 10.1210/en.2003-0644 12960020

[42] Mitchell JC Li XF Breen L Thalabard JC O’Byrne KT . The role of the locus coeruleus in corticotropin-releasing hormone and stress-induced suppression of pulsatile luteinizing hormone secretion in the female rat. Endocrinology. (2005) 146:323–31. doi: 10.1210/en.2004-1053 15486230

[43] D’Amelio N Gaggelli E Gajewska A Kochman H Kochman K Kozlowski H . Structural analysis and sheep pituitary receptor binding of GnRH and its complexes with metal ions. J Inorg Biochem. (2003) 94:28–35. doi: 10.1016/S0162-0134(02)00630-X 12620670

[44] Kochman K Kochman H Gajewska A Kozlowski H Nakamura K El-Mehasseb IM . (2001). “ Binding study of Cu, Ni and Co–GnRH complexes with the ovine pituitary receptor”, in: Proceedings of the VII International Symposium on Inorganic Biochemistry, Wroclaw, September 20-23, 2001, Abstract. Wrocław 35.

[45] Blitek A Ziecik A Gajewska A Kodaka M Counis R Kochman K . Cobalt complex with GnRH stimulates the LH release and PKA signaling pathway in pig anterior pituitary cells *in vitro*. Brain Res Bull. (2005) 65:391–6. doi: 10.1016/j.brainresbull.2005.02.008 15833593

[46] Rachamalla M Salahinejad A Kodzhahinchev V Niyogi S . Reproductive and developmental effects of sex-specific chronic exposure to dietary arsenic in zebrafish (Danio rerio). Toxics. (2024) 12:302. doi: 10.3390/toxics12040302 38668525 PMC11053724

[47] Feuer G Roomi MW Stuhne-Sekalec L Cameron RG . Association between progesterone binding and cytochrome P-450 content of hepatic microsomes in the rat treated with cobalt-haem. Xenobiotica. (1985) 15:407–12. doi: 10.3109/00498258509045011 4036166

[48] De Palma G Ortiz A Apostoli P . Effects of metallic elements on reproduction and development. In: Handbook on the toxicology of metals. Academic Press, Cambridge, MA, USA (2002). p. 565–92.

[49] Jana K Jana S Samanta PK . Effects of chronic exposure to sodium arsenite on hypothalamo-pituitary-testicular activities in adult rats: possible an estrogenic mode of action. Reprod Biol Endocrinol. (2006) 4:9. doi: 10.1186/1477-7827-4-9 16483355 PMC1397838

[50] Kamel FA Kubajak CL . Modulation of gonadotrophin secretion by corticosterone interaction with gonadal steroids and mechanism of action. Endocrinology. (1987) 121:561–5. doi: 10.1210/endo-121-2-561 3109884

[51] Wahli W Dawid IB Ryffel GU Weber R . Vitellogenesis and the vitellogenin gene family. Science. (1981) 212:298–304. doi: 10.1126/science.7209528 7209528

[52] Denslow ND Chow MC Kroll KJ Green L . Vitellogenin as a biomarker of exposure for estrogen or estrogen mimics. Ecotoxicology. (1999) 8:385–98. doi: 10.1023/a:1008986522208 41886696

[53] Momeni HR Eskandari N . Effect of vitamin E on sperm parameters and DNA integrity in sodium arsenite-treated rats. Iranian J Reprod Med. (2012) 10:249–56. PMC4165969 25243001

[54] Uckun FM Liu XP Cruz OJD . Human sperm immobilizing activity of aminophenyl arsenic acid and its N-substituted quinazoline, pyrimidine, and purine derivatives: Protective effect of glutathione. Reprod Toxicol. (2002) 16:57–64. doi: 10.1016/s0890-6238(01)00195-2 11934532

[55] Das J Ghosh J Manna P Sinha M Parames SC . Taurine protects rat testes against NaAsO2-induced oxidative stress and apoptosis via mitochondrial dependent and independent pathways. Toxicol Lett. (2009) 187:201–10. doi: 10.1016/j.toxlet.2009.03.001 19429265

[56] Yang L Mei G Yang Y Cui J Peng S Peng Z . Hexachlorocyclohexane impairs human sperm motility by affecting lysine glutarylation and mitochondrial functions. Food Chem Toxicol. (2023) 179:113991. doi: 10.1016/j.fct.2023.113991 37595880

[57] Zhang J Liu J Ren L Wei J Duan J Zhang L . PM(2.5) induces male reproductive toxicity via mitochondrial dysfunction, DNA damage and RIPK1 mediated apoptotic signaling pathway. Sci Tot Environ. (2018) 634:1435–44. doi: 10.1016/j.scitotenv.2018.03.383 29710643

[58] Collodel G Moretti E Noto D Iacoponi F Signorini C . Fatty acid profile and metabolism are related to human sperm parameters and are relevant in idiopathic infertility and varicocele. Mediat Inflammation. (2020) 31:3640450. doi: 10.1155/2020/3640450 32934603 PMC7479464

[59] Aksoy Y Aksoy H Altinkaynak K Aydin HR Ozkan A . Sperm fatty acid composition in subfertile men. Prostaglandins Leukot Ess Fat Acids. (2006) 75:75–9. doi: 10.1016/j.plefa.2006.06.002 16893631

[60] Marzec-Wroblewska U Kaminski P Łakota P Szymanski M Wasilow K Ludwikowski G . Human sperm characteristics with regard to cobalt, chromium, and lead in semen and activity of catalase in seminal plasma. Biol Trace Elem Res. (2011) 188:51–260. doi: 10.1007/s12011-018-1416-9 29959647

[61] Kumar GP Laloraya M Laloraya MM . Powerful anti-sperm motility action of cobaltous ion and its recovery by a sulfhydryl compound. Contraception. (1990) 41:633–9. doi: 10.1016/s0010-7824(09)91008-3 2163298

